# Supplementary material for: Lola-I is a promoter pioneer factor that establishes de novo Pol II pausing during development
Source: Nat Commun. 2023 Sep 21;14:5862. doi: 10.1038/s41467-023-41408-1 (PMC10514308; doi:10.1038/s41467-023-41408-1)
Supplement: Supplementary file 1 — Supplementary Information [file 41467_2023_41408_MOESM1_ESM.pdf]

Supplementary Figures

Supplementary Fig. 1

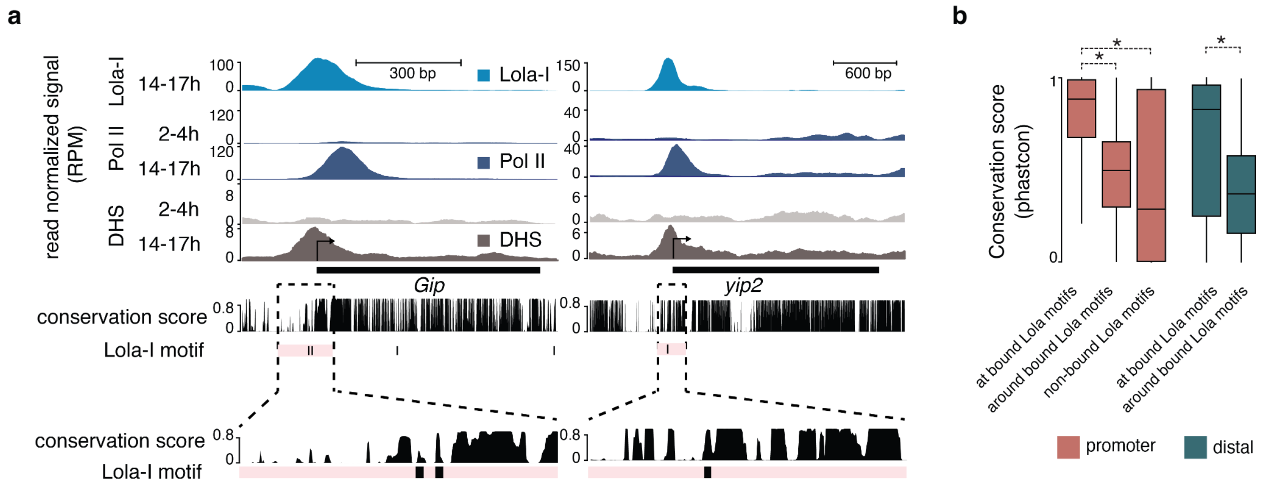

Supplementary Fig. 1: Conservation of Lola-I motifs. **a**) Single-gene examples of Lola-I target promoters showing Lola-I binding, Pol II binding, DNase hypersensitivity (DHS) and the conservation of Lola-I binding sites across different *Drosophila* species using the PhastCons score. RPM: normalized reads per million. **b**) Boxplots of PhastCons scores at Lola-I motifs show significant conservation of bound Lola-I motifs. Bound Lola-I motifs at promoters are highly conserved compared to promoter regions in general (100 bp centered on the motif) or non-bound Lola-I motifs in promoters (Wilcoxon two-sided test, *P <2e-16). Similarly, Lola-I binding sites at distal (non-promoter) regions bound by Lola-I are also highly conserved (Wilcoxon two-sided test, *P <2e-16).

Supplementary Fig. 2

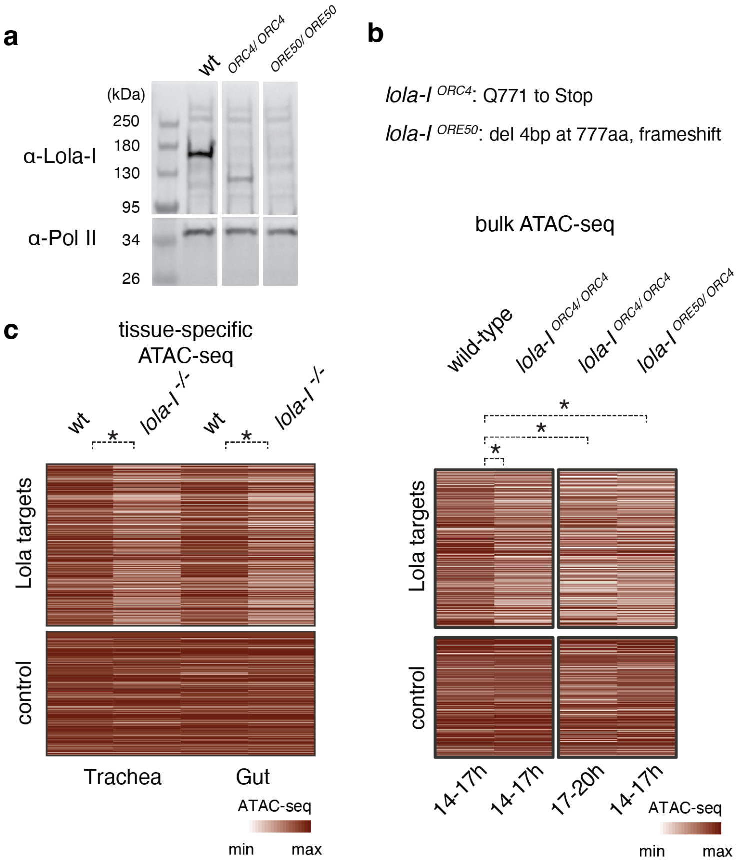

Supplementary Fig. 2: Genomic characterization of *lola-I* mutants: **a**) Mutant line *lola-I* ^{ORC4} [29] has a premature stop codon before the C2H2 zinc-finger region that codes for the DNA binding domain. Mutant line *lola-I* ^{ORE50} [29] has a frame-shift mutation that leads to degradation of the Lola-I protein product. The Rpb3 subunit of Pol II is shown as control below. The shown lanes were not run adjacently in the original gel. kDa: kilodalton. A single biological experiment was performed but the results are consistent with other orthogonal experiments. Source data is provided as a Source Data file. **b**) Heatmap showing ATAC-seq accessibility at Lola-I targets and control promoters for wild-type and mutant combinations. Lola-I targets show reduced accessibility in *lola*^{ORC4} homozygous mutant embryos over wild-type at 14-17h (Wilcoxon two-sided test, *P = 2.2e-10) and this extends to 17-20h (Wilcoxon two-sided test, *P = 3.4e-11), confirming that the opening of these promoters is not just delayed in *lola-I* mutants. Importantly, trans-heterozygous *lola* $^{ORE50/ORC4}$ mutant embryos (14-17h) show a similar reduction in accessibility compared to homozygous *lola* $^{ORC4/ORC4}$ embryos (Wilcoxon two-sided test, *P = 1.2e-14), demonstrating that the reduced accessibility is due to the mutation in *lola-I* and not due to other mutations in the *lola* ^{ORC4} line. *lola* ^{ORC4} mutant has a premature stop codon before the DNA binding domain. *lola*^{ORE50}mutant has a frameshift mutation before the DNA binding domain. **c**) Heatmap showing reduced ATAC-seq accessibility at Lola-I targets in *lola* $^{-/-}$ mutant embryos over wild-type at 14-17h when tracheal nuclei (left) or gut nuclei (right) were isolated using the INTACT method (Wilcoxon two-sided test, Trachea = 1.5e-11; Gut = *P = 1.9e-9). These tissues were selected because the driver lines expressing nuclear-envelope BirA in these tissues are located on the second chromosome and thus could be recombined with the *lola-I* mutants. The results show that the reduced ATAC-seq accessibility of Lola-I target promoters in *lola-I* mutants is not tissue-specific.

Supplementary Fig. 3

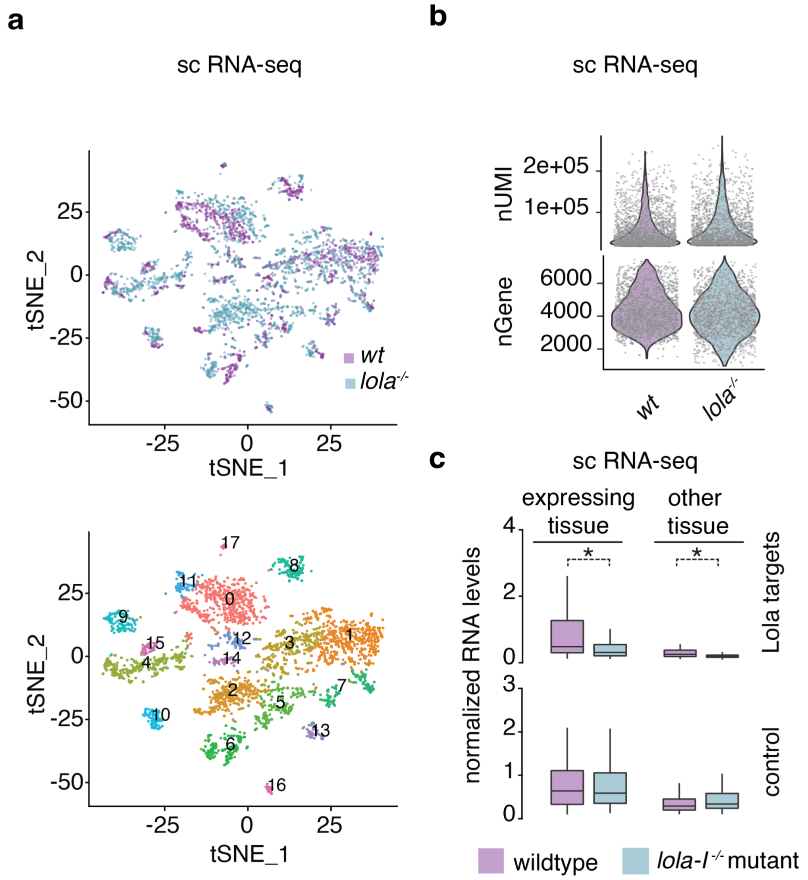

Supplementary Fig. 3: scRNA-seq analysis of *lola-I* mutants: **a**) Single-cell RNA-seq (scRNA-seq) tSNE map of cells from wild-type and *lola-I* mutant homozygous embryos are shown. Top panel: the projection of the scRNA-seq data shows alignment between the wild-type and *lola-I* mutant cells. Bottom panel: the identified clusters in these scRNA-seq data show that wild-type and *lola-I* mutant cells cluster together. **b**) Number of Unique Molecular identifiers (UMI) and number of genes captured per cell are comparable between the wild-type and *lola-I* mutant samples, indicating similar data quality. **c**) Boxplots of scRNA-seq data show that both the tissue-specific expression and the basal expression of Lola-I targets (at least a two-fold change in pol II occupancy between wild-type and mutant embryos) are reduced in the *lola-I* embryos compared to wild-type embryos (both profiled at 14-14.5h (Wilcoxon two-sided test, expressing tissue - *P = 0.019, other tissues - *P = 0.0032, adjustment method: holm). Normalized expression in each cell shown. Box plots in B and G show the median as the central line, the first and the third quartiles as the box, and the upper and lower whiskers extend from the quartile box to the largest/smallest value within 1.5 times of the interquartile range.

Supplementary Fig. 4

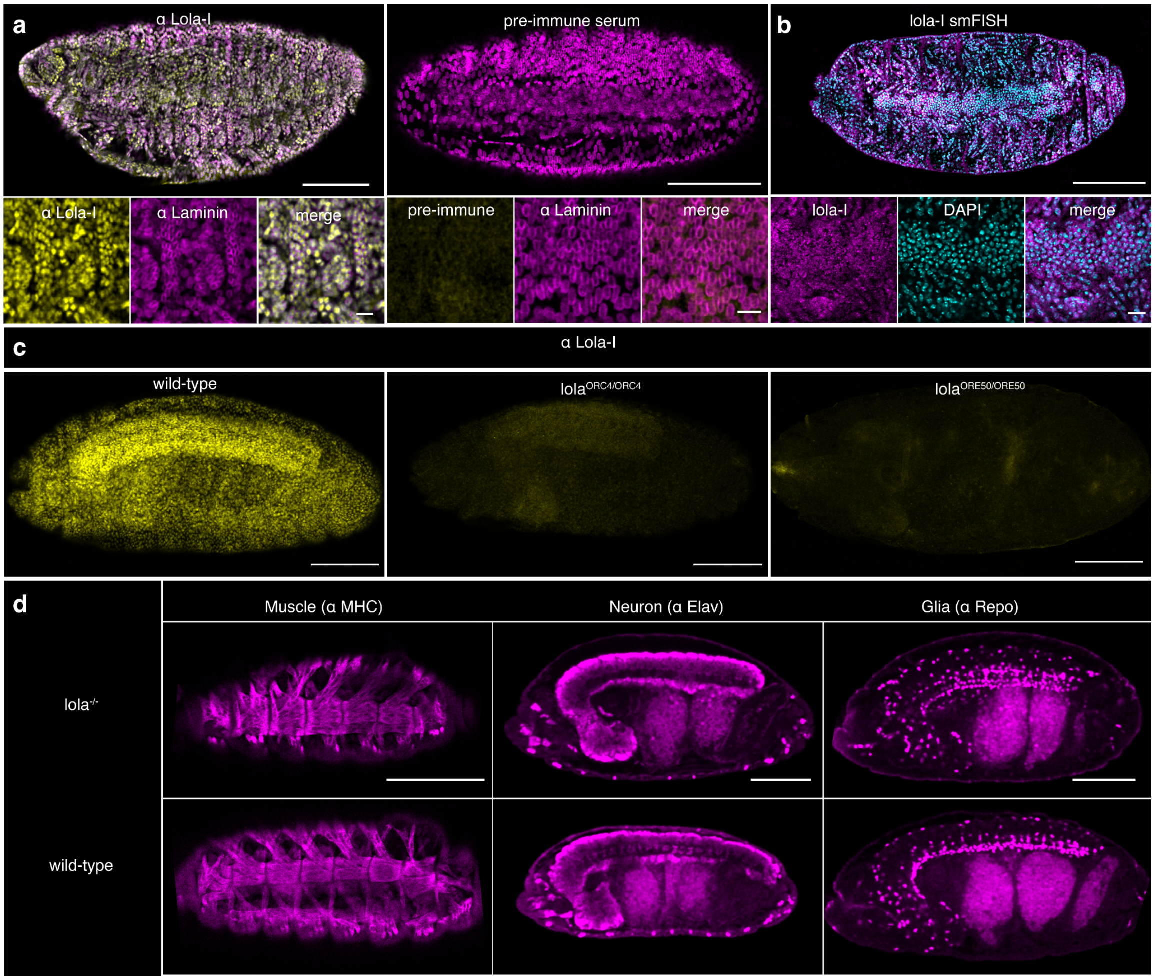

Supplementary Fig. 4: Immunostainings showing ubiquitous Lola-I expression and a *lola-I* mutant phenotype that appears anatomically normal.

a) Immunostainings using antibodies against Lola-I (1:750) and Lamin (1:750) as control show ubiquitous nuclear signals of Lola-I throughout the embryo (left). Control Immunostainings with the pre-immune serum from the Lola-I antibody production shows no nuclear signal (right). scale bar: top - 100 µm and bottom - 10 µm. Results shown are representative of at least two biological replicates and many embryos and are consistent. **b**) Single-molecule FISH also shows ubiquitous expression of *lola-I* transcripts. scale bar: top - 100 µm and bottom - 10 µm). Results shown are representative of at least two biological replicates and many embryos and are consistent. **c**) *lola-I* mutants show marked decrease in signal for the Lola-I antibody immunostaining compared to the wildtype embryos. Immunostainings were done using antibodies against Lola-I (1:750). scale bar: 100 µm. Results shown are representative of a single biological experiment but across multiple embryos and are consistent. **d**) *lola-I* mutants do not show any gross visible phenotypic defects but fail to hatch. Embryos were immunostained to visualize muscle (alpha-MHC antibodies), neurons (Elav antibodies) and glia (Repo antibodies), respectively. scale bar: 100 µm. For all images here the contrast was adjusted linearly in every image and set the same in panel A (overviews and zooms) for **α** Lola-I and pre-immune serum and separately for **α** Laminin. Additionally, the lookup tables for all images are linear. The settings in the individual panels are the same as in the merge. Results shown are representative of at least two biological replicates and many embryos and are consistent.

Supplementary Fig. 5

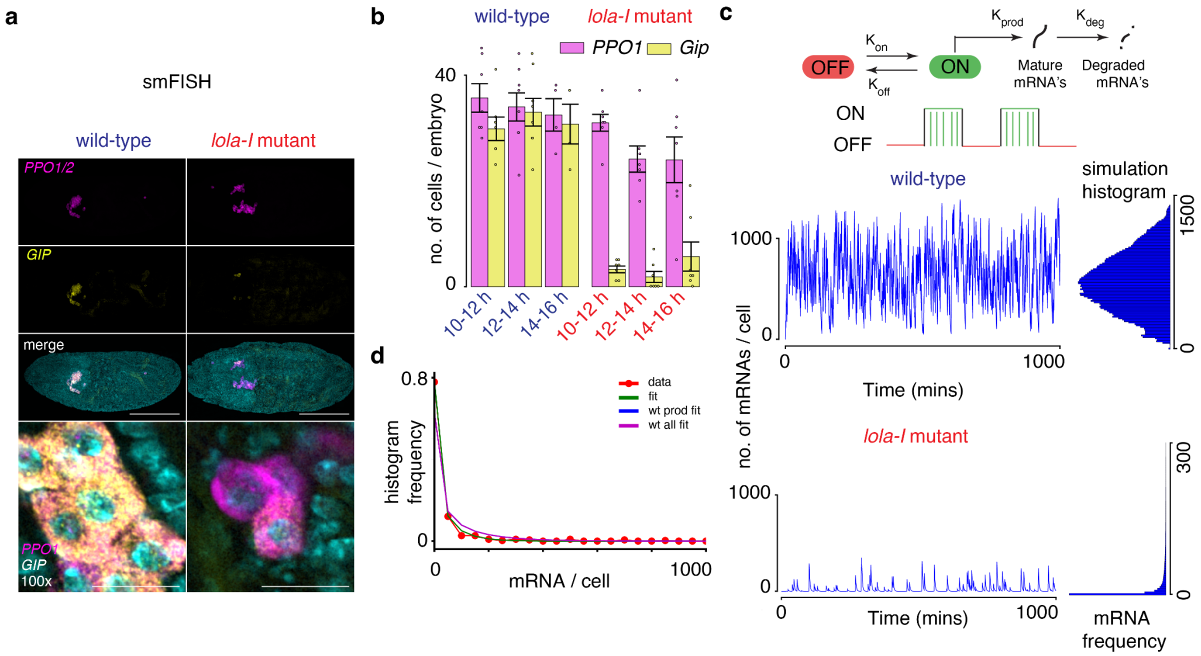

Supplementary Fig. 5: Quantification of expression changes in Lola-I mutants using single-molecule FISH. **a**) Example of single-molecule FISH images of *Gip* (yellow), and *PPO1* and *PPO2* genes (pink) in wild-type (wt) embryos and *lola-I* mutant embryos (blue - DAPI). Data were acquired with 10x magnification (top three panels); scale bar = 100 µm and 100x magnification (bottom panel); scale bar = 20 µm. Here the images were all brightness and contrast adjusted for clarity and linearly. The lookup tables for each image are linear. The settings in the individual panels are the same as in the merge. Results shown are representative of at least two biological replicates and many embryos and are consistent. **b**) The number *Gip*-positive cells are strongly reduced in *lola-I* mutant embryos compared to wild-type, while the *PPO1*/*PPO2* positive cells remain similar. Bar plots show data for wild-type: 10-12 h n=7, 12-14 h n=8, 14-16 h n=4, *lola-I*mutant: 10-12 h n=7, 12-14 h n=7, 14-16 h n=7. Average is shown as a bar with standard error of the mean as error bars. Reduced *Gip* expression is observed for multiple time-points showing that it is not due to a developmental delay and that *Gip* expression only mildly recovers over time. Source data is provided as a Source Data file. **c**) Simulations of bursty transcription for a single cell (left) and the histogram of mRNA levels (right) for wt fit parameters (top row) and mutant fit parameters (bottom row). The model of transcription used in the simulations and fits of experimental histograms. Gene activation (with rate constant, K_{on}) leads to the initiation of a mRNA production burst with the appearance of nascent spots. Gene inactivation (with a rate constant, K_{off}) causes the disappearance of nascent spots and leads to the decay of mature mRNA levels (due to degradation) at the end of a burst. Wild-type bursts are so frequent that one rarely finds dark cells, while mutant bursts are infrequent, leading to mostly dark cells. **d**) Distribution of the numbers of mRNAs per cell quantified from the total fluorescence intensity (divided by the intensity of a single RNA molecule) of the single-molecule FISH signal from each cell in the *lola-I* mutant embryos (yellow dots). Here, many *PPO1/PPO2* positive cells were dark for *Gip* signal. The distribution was fit according to the two-state model (see methods). In one case the data was fit with all parameters allowed to converge (green line). In another case the data was fit with the production rate fixed to wild-type while all other parameters were allowed to converge (blue line). In the final case, all adjustable parameters were fixed to the wild-type values (purple line).

Supplementary Fig. 6

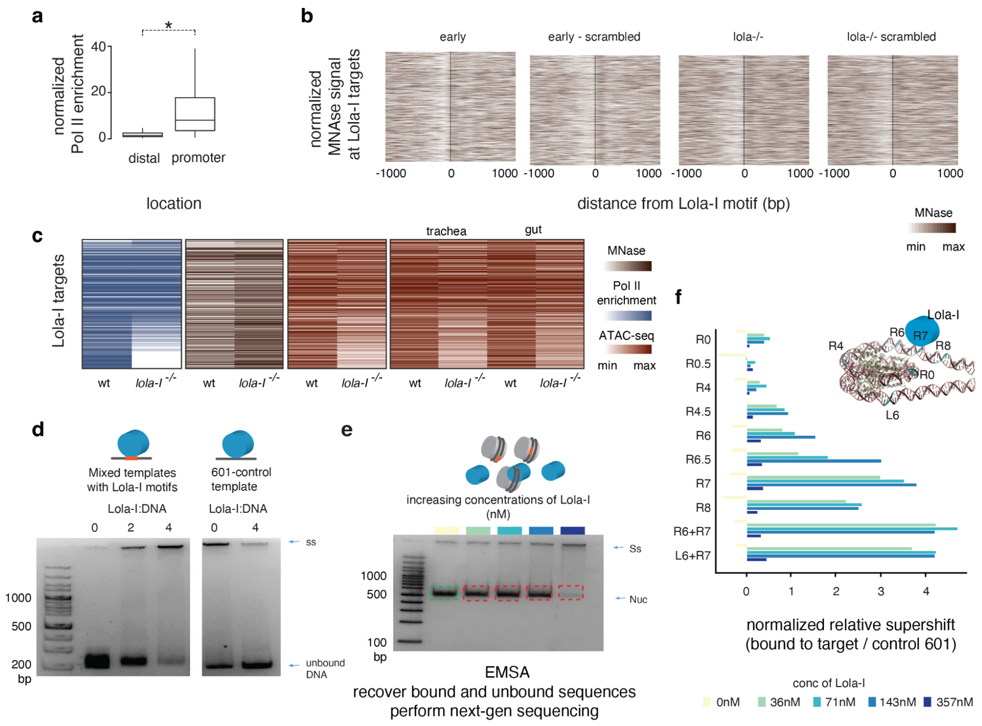

Supplementary Fig. 6: Additional information on in vitro nucleosome binding assays. **a**) Box plot shows normalized Pol II enrichment (wt 14-17 h) at both the promoter proximal and distal Lola-I peak regions. Pol II signal is found only at the annotated promoter regions and not at the distal Lola-I target regions (Wilcoxon two-sided test, *P <2e-16). Box plot shows the median as the central line, the first and the third quartiles as the box, and the upper and lower whiskers extend from the quartile box to the largest/smallest value within 1.5 times of the interquartile range. **b**) Nucleosome occupancy centered on Lola-I motifs or randomly scrambled (-85 or +85 bp) are plotted as MNase-seq heatmap at each Lola-I binding site for the early (2-4 h) wt or late (14-17 h) Lola-I mutant samples. The left-right orientation is chosen such that the nucleosomes on either side of the motif align. The results suggest that the Lola-I motifs might be preferentially found along the nucleosomal edges in vivo. **c**) Heatmap showing increased nucleosome occupancy, reduced Pol II, whole embryo ATAC-seq and ATAC-seq accessibility at Lola-I targets in tracheal or gut tissues in *lola* $^{-/-}$ mutant embryos over wild-type at 14-17h. In general, Lola-I targets that show reduced Pol II occupancy, also show reduced chromatin accessibility in the mutants. All the Heatmaps are sorted by the change in Pol II signal between the wt and mutant embryos. **d**) Binding of Lola-I to the 601-templates with Lola-I motif (left panel) or 601-control template without any Lola-I motif (left panel) at different concentrations of Lola-I is measured. Lola-I binds to the 601-templates with the Lola-I motifs at lower concentration than to the 601-control template. bp: base pairs. Results shown are representative of at least two comparable biological experiments and are consistent. bp: base pairs. Source data is provided as a Source Data file. **e**) The gel-shift assay shows the binding of Lola-I to nucleosome bound 601 sequences with differing Lola-I motifs. Both the nucleosomal and the super-shifted fractions are purified and sequenced to measure the relative affinity of Lola-I to different Lola-601 sequences. Results shown are representative of at least two biological replicates and are consistent. bp: base pairs. Source data is provided as a Source Data file. **f**) Relative super-shift for Lola-I binding at different concentrations to nucleosomes with 601-Lola-I sequences with Lola-I motif vs control-601 template without the Lola-I motif (relative to the no Lola-I lane), are shown. Lola-I strongly binds when the motif is located along the nucleosomal edge. Results shown are a summary of at least two biological replicates and are consistent. At the highest Lola-I concentration, non-specific binding occurs, which reduces the relative super-shift values. The nucleosome structure is from the RSCB protein data bank (5NL0).
