# Supplementary material for: Lola-I is a promoter pioneer factor that establishes de novo Pol II pausing during development
Source: Nat Commun. 2023 Sep 21;14:5862. doi: 10.1038/s41467-023-41408-1 (PMC10514308; doi:10.1038/s41467-023-41408-1)
Supplement: Supplementary file 3 — Description of Additional Supplementary Files [file 41467_2023_41408_MOESM3_ESM.pdf]

Description of Additional Supplementary Files

Supplementary Data 1:

The supplementary data sheet shows the list of motifs that are enriched or depleted in the opening set genes relative to the constant set genes based on the chi-squared test after correcting for multiple testing by Benjamini-Hochberg (BH) correction method.

Supplementary Data 2:

The sheet 1 shows the list of Lola-I target and control non-target promoters. The sheet 2 shows the list of Lola-I peaks that are in the promoter or distal regions and their coordinates.

Supplementary Data 3:

The sheet shows various Bioprocess GO terms that are enriched for the Lola-I target genes using a hypergeometric test (one-sided - over enrichment) with no multiple testing correction.

Supplementary Data 4:

The calculation of various transcription model parameters was calculated as a ratio to the degradation rate constant that is not known. The sheet shows the various model parameters if we assume degradation half-lives to be 20, 75 and 150 mins, which are within reasonable limits.

Supplementary Data 5:

The supplementary data shows pearson correlations between various samples and replicates. Chipseq, atacseq, mnaseq sample correlations were calculated based on the signals at all promoters. RNAseq correlations were calculation based on gene expression.
