# Supplementary material for: Lola-I is a promoter pioneer factor that establishes de novo Pol II pausing during development
Source: Nat Commun. 2023 Sep 21;14:5862. doi: 10.1038/s41467-023-41408-1 (PMC10514308; doi:10.1038/s41467-023-41408-1)
Supplement: Supplementary file 9 — Reporting Summary [file 41467_2023_41408_MOESM9_ESM.pdf]

nature portfolio

Corresponding author(s): Julia Zeitlinger

Last updated by author(s): Jul 21, 2022

Reporting Summary

Nature Portfolio wishes to improve the reproducibility of the work that we publish. This form provides structure for consistency and transparency in reporting. For further information on Nature Portfolio policies, see our Editorial Policies and the Editorial Policy Checklist.

Statistics

For all statistical analyses, confirm that the following items are present in the figure legend, table legend, main text, or Methods section.

n/a	Confirmed	
☐	☒	The exact sample size (*n*) for each experimental group/condition, given as a discrete number and unit of measurement
☐	☒	A statement on whether measurements were taken from distinct samples or whether the same sample was measured repeatedly
☐	☒	The statistical test(s) used AND whether they are one- or two-sided<br>*Only common tests should be described solely by name; describe more complex techniques in the Methods section.*
☒	☐	A description of all covariates tested
☐	☒	A description of any assumptions or corrections, such as tests of normality and adjustment for multiple comparisons
☐	☒	A full description of the statistical parameters including central tendency (e.g. means) or other basic estimates (e.g. regression coefficient) AND variation (e.g. standard deviation) or associated estimates of uncertainty (e.g. confidence intervals)
☐	☒	For null hypothesis testing, the test statistic (e.g. *F*, *t*, *r*) with confidence intervals, effect sizes, degrees of freedom and *P* value noted<br>*Give P values as exact values whenever suitable.*
☒	☐	For Bayesian analysis, information on the choice of priors and Markov chain Monte Carlo settings
☒	☐	For hierarchical and complex designs, identification of the appropriate level for tests and full reporting of outcomes
☐	☒	Estimates of effect sizes (e.g. Cohen's *d*, Pearson's *r*), indicating how they were calculated

Our web collection on statistics for biologists contains articles on many of the points above.

Software and code

Policy information about availability of computer code

Data collection	ChIP-seq data and ATAC-seq experiments were aligned using Bowtie (1.1.2)<br>Bulk mRNAseq samples, pseudo-alignment was performed using the Kallisto package (0.46.0)<br>scRNA-seq samples, alignment and separations of reads from different cells and quantification of gene expression were done using the Cell Ranger pipeline (2.1.1)<br>R - 3.6.1
Data analysis	All data analysis performed in this paper, including raw data, processed data, software tools, and analysis scripts are available through a publicly accessible Amazon Linux virtual machine image (ami-id: ami-013fa11a4a9b52628)(https://aws.amazon.com/console/). The analysis code is also available on GitHub at https://github.com/zeitlingerlab/Ramalingam_Lola_2022.git. The analysis code is also available on Zotero.

For manuscripts utilizing custom algorithms or software that are central to the research but not yet described in published literature, software must be made available to editors and reviewers. We strongly encourage code deposition in a community repository (e.g. GitHub). See the Nature Portfolio guidelines for submitting code & software for further information.

Data

Policy information about availability of data

All manuscripts must include a data availability statement. This statement should provide the following information, where applicable:

- Accession codes, unique identifiers, or web links for publicly available datasets
- A description of any restrictions on data availability
- For clinical datasets or third party data, please ensure that the statement adheres to our policy

Raw and processed functional genomics data associated with this manuscript have been deposited in GEO under accession number, “GSE200875 [https://

www.ncbi.nlm.nih.gov/geo/query/acc.cgi?acc=GSE200875]". Part of the wild-type functional genomics data is available from the previously released GEO dataset, "GSE120157 [https://www.ncbi.nlm.nih.gov/geo/query/acc.cgi?acc=GSE120157]". Source data for the graphs are provided with this paper. The nucleosome model figure uses the nucleosome structure from the RCSB PDB database, "5NL0, [https://www.rcsb.org/structure/5nl0]". The raw and processed data are also available through a publicly accessible Amazon Linux virtual machine image (ami-id: ami-013fa11a4a9b52628) (https://aws.amazon.com/console/) and the unprocessed microscopy and western blot images are available through the stowers institute's original data repository (https://www.stowers.org/stowers-odr).

Field-specific reporting

Please select the one below that is the best fit for your research. If you are not sure, read the appropriate sections before making your selection.

☒ Life sciences ☐ Behavioural & social sciences ☐ Ecological, evolutionary & environmental sciences

For a reference copy of the document with all sections, see nature.com/documents/nr-reporting-summary-flat.pdf

Life sciences study design

All studies must disclose on these points even when the disclosure is negative.

Sample size	No statistical method was used to predetermine sample size. Sample size of at least 2 biological replicates were used for of ChIP-seq, RNA-seq and scRNA-seq, and ATAC-seq experiments as is a common practice in the field. Sample size of at least 2 biological replicates were used for key Western Blot and Immunostaining experiments.<br>smFISH and in-vitro binding assay was done in duplicates..<br>MNAse-seq in wild-type conditions (2-4 hrs) does not include replicates as it involved analysis of previously published data. However the results are consistent with other unrelated datasets.
Data exclusions	Replicates of scRNAseq were excluded from the analysis, as they were done with different single-cell chemistry or with different PCR cycling conditions. It is is not clear in the field how to properly align different scRNAseq datasets without over-correcting for batch effects. We note in the text the limitations of analyzing low gene expression with scRNAseq analysis. The main conclusions from the scRNAseq data, however, are consistent across replicates. When several replicates are present, for simplicity, replicates with higher read depth or replicates that allow for side-by-side comparisons are chosen.
Replication	Replicates consistency was measured using pearson correlation. As shown in Supplementary Data 5, the replicates show very good correlations and attempts at reproduction of the results were successful.
Randomization	This research does not involve randomization of samples.
Blinding	Blinding was not performed due to the unambiguous nature of measurements and systematic analyses used in these experiments.

Reporting for specific materials, systems and methods

We require information from authors about some types of materials, experimental systems and methods used in many studies. Here, indicate whether each material, system or method listed is relevant to your study. If you are not sure if a list item applies to your research, read the appropriate section before selecting a response.

Materials & experimental systems

n/a	Involved in the study
☐	☒ Antibodies
☐	☒ Eukaryotic cell lines
☒	☐ Palaeontology and archaeology
☐	☒ Animals and other organisms
☒	☐ Human research participants
☒	☐ Clinical data
☒	☐ Dual use research of concern

Methods

n/a	Involved in the study
☐	☒ ChIP-seq
☒	☐ Flow cytometry
☒	☐ MRI-based neuroimaging

Antibodies

Antibodies used	Lola-I (custom-made by Genescript), α-tubulin antibodies (Sigma, T5168), Lamin (ADL40 from Developmental Studies Hybridoma Bank, DSHB, at 1:750), α-MHC (source not tractable but validated by immuno-staining experiments; also these are only used in the supplements to support a minor point), Elav (7E8A10 from DSHB), Repo (8D12 from DSHB), Pol II (Rpb3, custom made from GeneScript, Zeitlinger lab 163185-50)
Validation	The Lola-I antibody was validated by the presence of a correctly sized band in Western blots and by the loss of this band in the Lola-I mutant. In addition, ChIP-seq experiments in wild-type revealed the known 6-mer motif (AAAGCT) that Lola-I binds. Lola-I antibody was also validated by the loss signal in immuno-staining assays in the Lola-I mutants.Other antibodies were previously validated. α-tubulin was verified using western blot by the company. Lamin, α-MHC ,Elav, Repo antibodies were validated by immuno-staining

patterns consistent with the target protein expression from independent studies. Rpb3 antibodies were validated by western blot and several previously published ChIP-seq experiments.

Eukaryotic cell lines

Policy information about cell lines

Cell line source(s)	The Sf9 insect cell line is a clonal isolate derived from the parental Spodoptera frugiperda cell line IPLB-Sf-21-AE.
Authentication	No known authentication of the cell line was performed
Mycoplasma contamination	No known testing for mycoplasma contamination was performed
Commonly misidentified lines (See ICLAC register)	None of the listed misidentified lines were used.

Animals and other organisms

Policy information about studies involving animals; ARRIVE guidelines recommended for reporting animal research

Laboratory animals	Oregon-R was used as the wild-type strain. Lola-I mutant lines were obtained from Bloomington stock center (ORC4 - 28267) and from Edward Giniger (ORE50). Homozygous lola-I mutant files were non-viable and were maintained over a CyO-GFP balancer to allow sorting of the homozygous mutant embryos that are GFP-. Lola-I rescue lines were generated as follows: a construct with an Actin promoter driving full-length lola-I cDNA and marked by mini-white was integrated into the attP40 locus on 2L and then crossed with the lola-I ORC4 line (lola is on 2R) to obtain females that recombine the second chromosome in the germ line. After crossing in a CyO balancer, several males harboring the mini-white marker were selected. After mating single males with a CyO balancer stock, each male was screened for the lola-I ORC4 mutation by amplifying the relevant portion of the lola locus by PCR and sequencing. Meiotic recombinants that had both the rescue construct and the lola-I ORC4 mutation were viable as homozygotes. For the INTACT experiments, embryos from fly stocks expressing tissue-specific RAN-GAP-mcherry-FLAG-BirA with the help of tissue-specific Gal4 driver lines were collected as described (Ramalingam et al., 2021). To isolate tracheal or gut cells from lola -/- embryos, fly lines containing RAN-GAP-mcherry-FLAG-BirA on the second chromosome (expressing in either trachea or gut) was recombined with the lola-I ORC4 chromosome and maintained over a GFP-marked CyO balancer (Trachea: w[*]; P{w[+mC]=GAL4-btl.S}2, P{w[+m*]=lacZ-un8}276, p[UAS-3xFLAG-blrp-mCherry-RanGap, UAS-BirA)5; lolaorc4/CyO-GFP) (Gut: w[*]; P{GawB}NP3084 , p[UAS-3xFLAG-blrp-mCherry-RanGap, UAS- BirA)5; lolaorc4/CyO-GFP). Homozygous embryos for the recombinant chromosome were obtained by sorting for GFP-negative embryos.
Wild animals	No wild animals were used in this study
Field-collected samples	This study did not involve field collected samples.
Ethics oversight	No ethical approval is required for this study involving Drosophila. The Lola-I antibodies and protein were raised in rabbit commercially by Genscript according the company's ethical protocols.

Note that full information on the approval of the study protocol must also be provided in the manuscript.

ChIP-seq

Data deposition

☒ Confirm that both raw and final processed data have been deposited in a public database such as GEO.

☒ Confirm that you have deposited or provided access to graph files (e.g. BED files) for the called peaks.

Data access links *May remain private before publication.*	All raw and processed data have been deposited in GEO under session number GSE200875 (access_token: klkzuacwbhytrsv, url: https://www.ncbi.nlm.nih.gov/geo/query/acc.cgi?acc=GSE200875). All data analysis performed in this paper, including raw data, processed data, software tools, and analysis scripts are available through publicly accessible Amazon Linux virtual machine image (ami-id: ami-08731b3f99f24143f). Part of wildtype data is also accessible through the previously released GEO submission (GSE120157).
Files in database submission	GSM6045681 ATAC-seq in wildtype INTACT Gut 14-17hrs embryo Rep1<br>GSM6045682 ATAC-seq in wildtype INTACT Gut 14-17hrs embryo Rep2<br>GSM6045683 ATAC-seq in wildtype INTACT Gut 14-17hrs embryo Rep3<br>GSM6045684 ATAC-seq in wildtype INTACT Epidermis 14-17hrs embryo Rep1<br>GSM6045685 ATAC-seq in wildtype INTACT Epidermis 14-17hrs embryo Rep2<br>GSM6045686 ATAC-seq in wildtype INTACT Muscle 14-17hrs embryo Rep1<br>GSM6045687 ATAC-seq in wildtype INTACT Muscle 14-17hrs embryo Rep2<br>GSM6045688 ATAC-seq in wildtype INTACT Neuron 14-17hrs embryo Rep1<br>GSM6045689 ATAC-seq in wildtype INTACT Neuron 14-17hrs embryo Rep2<br>GSM6045690 ATAC-seq in wildtype INTACT Trachea 14-17hrs embryo Rep1<br>GSM6045691 ATAC-seq in wildtype INTACT Trachea 14-17hrs embryo Rep2<br>GSM6045692 ATAC-seq in wildtype INTACT Trachea 14-17hrs embryo Rep3<br>GSM6045693 ATAC-seq in Lola-I mutant ORC4 Gut 14-17hrs embryo Rep1<br>GSM6045694 ATAC-seq in Lola-I mutant ORC4 Gut 14-17hrs embryo Rep2<br>GSM6045695 ATAC-seq in Lola-I mutant ORC4 Trachea 14-17hrs embryo Rep1

GSM6045696 ATAC-seq in Lola-I mutant ORC4 Trachea 14-17hrs embryo Rep2
GSM6045697 ATAC-seq in Lola-I mutant ORC4 14-17hrs embryo Rep1
GSM6045698 ATAC-seq in Lola-I mutant ORC4 14-17hrs embryo Rep2
GSM6045699 ATAC-seq in Lola-I mutant ORC4 17-20hrs embryo Rep1
GSM6045700 ATAC-seq in Lola-I mutant ORC4 17-20hrs embryo Rep2
GSM6045701 ATAC-seq in Lola-I rescue 14-17hrs embryo Rep1
GSM6045702 ATAC-seq in Lola-I rescue 14-17hrs embryo Rep2
GSM6045703 ATAC-seq in Lola-I mutant transheterozygous ORE50/ORC4 14-17hrs embryo Rep1
GSM6045704 ATAC-seq in Lola-I mutant transheterozygous ORE50/ORC4 14-17hrs embryo Rep2
GSM6045705 ATAC-seq in wildtype OregonR 14-17hrs embryo Rep1
GSM6045706 ATAC-seq in wildtype OregonR 14-17hrs embryo Rep2
GSM6045721 Epidermis_7021_INTACT_Lola_I_14-17h_1
GSM6045722 Epidermis_7021_INTACT_Lola_I_14-17h_2
GSM6045723 Epidermis_7021_INTACT_Lola_I_14-17h_3
GSM6045724 Epidermis_7021_INTACT_Rpb3_14-17h_1
GSM6045725 Epidermis_7021_INTACT_Rpb3_14-17h_2
GSM6045726 Epidermis_7021_INTACT_WCE_14-17h_1
GSM6045727 Epidermis_7021_INTACT_WCE_14-17h_2
GSM6045728 Glia_repo_INTACT_Rpb3_14-17h_1
GSM6045729 Glia_repo_INTACT_Rpb3_14-17h_2
GSM6045730 Glia_repo_INTACT_WCE_14-17h_1
GSM6045731 Glia_repo_INTACT_WCE_14-17h_2
GSM6045732 Gut_110394_INTACT_Lola_I_14-17h_1
GSM6045733 Gut_110394_INTACT_Lola_I_14-17h_2
GSM6045734 Gut_110394_INTACT_Rpb3_14-17h_1
GSM6045735 Gut_110394_INTACT_Rpb3_14-17h_2
GSM6045736 Gut_110394_INTACT_WCE_14-17h_1
GSM6045737 Gut_110394_INTACT_WCE_14-17h_2
GSM6045738 Muscle_mef2_INTACT_Lola_I_14-17h_1
GSM6045739 Muscle_mef2_INTACT_Lola_I_14-17h_2
GSM6045740 Muscle_mef2_INTACT_Lola_I_14-17h_3
GSM6045741 Muscle_mef2_INTACT_Rpb3_14-17h_1
GSM6045742 Muscle_mef2_INTACT_Rpb3_14-17h_2
GSM6045743 Muscle_mef2_INTACT_WCE_14-17h_1
GSM6045744 Muscle_mef2_INTACT_WCE_14-17h_2
GSM6045745 Neuron_elav_INTACT_Lola_I_14-17h_1
GSM6045746 Neuron_elav_INTACT_Lola_I_14-17h_2
GSM6045747 Neuron_elav_INTACT_Lola_I_14-17h_3
GSM6045748 Neuron_elav_INTACT_Rpb3_14-17h_1
GSM6045749 Neuron_elav_INTACT_Rpb3_14-17h_2
GSM6045750 Neuron_elav_INTACT_WCE_14-17h_1
GSM6045751 Neuron_elav_INTACT_WCE_14-17h_2
GSM6045752 Trachea_8807_INTACT_Lola_I_14-17h_1
GSM6045753 Trachea_8807_INTACT_Lola_I_14-17h_2
GSM6045754 Trachea_8807_INTACT_Rpb3_14-17h_1
GSM6045755 Trachea_8807_INTACT_Rpb3_14-17h_2
GSM6045756 Trachea_8807_INTACT_WCE_14-17h_1
GSM6045757 Trachea_8807_INTACT_WCE_14-17h_2
GSM6045758 Trachea_8807_orc4_INTACT_Rpb3_14-17h_1
GSM6045762 Dme_emb_14-17h_Lola_I_1
GSM6045763 Dme_emb_14-17h_Lola_I_2
GSM6045764 Dme_emb_14-17h_Rpb3_1
GSM6045765 Dme_emb_14-17h_Rpb3_2
GSM6045766 Dme_emb_14-17h_Rpb3_3
GSM6045767 Dme_emb_14-17h_WCE_1
GSM6045768 Dme_emb_14-17h_WCE_2
GSM6045769 Dme_emb_14-17h_WCE_3
GSM6045773 Dme_emb_2-4h_Rpb3_1
GSM6045774 Dme_emb_2-4h_Rpb3_2
GSM6045775 Dme_emb_2-4h_Rpb3_3
GSM6045776 Dme_emb_2-4h_WCE_1
GSM6045777 Dme_emb_2-4h_WCE_2
GSM6045778 Dme_emb_2-4h_WCE_3
GSM6045779 dme_emb_act_Lola_I_orc4_14-17h_Rpb3_1
GSM6045780 dme_emb_act_Lola_I_orc4_14-17h_Rpb3_2
GSM6045781 dme_emb_act_Lola_I_orc4_14-17h_WCE_1
GSM6045782 dme_emb_act_Lola_I_orc4_14-17h_WCE_2
GSM6045786 Dme_emb_orc4_14-17h_Rpb3_1
GSM6045787 Dme_emb_orc4_14-17h_Rpb3_2
GSM6045788 Dme_emb_orc4_14-17h_Rpb3_3
GSM6045789 Dme_emb_orc4_14-17h_WCE_1
GSM6045790 Dme_emb_orc4_14-17h_WCE_2
GSM6045791 Dme_emb_orc4_14-17h_WCE_3
GSM6045792 Dme_MNase_14-17h_1
GSM6045793 Dme_MNase_14-17h_2
GSM6045794 Dme_MNase_2-4h_1

GSM6045795 Dme_MNase_orc4_14-17h_1
GSM6045796 Dme_MNase_orc4_14-17h_2
GSM6045809 mrna_wt_14to17h_1
GSM6045810 mrna_wt_14to17h_2
GSM6045811 mrna_wt_14to17h_3
GSM6045812 mrna_wt_14to17h_5
GSM6045813 mrna_wt_14to17h_6
GSM6045814 mrna_ORC4_14to17h_1
GSM6045815 mrna_ORC4_14to17h_2
GSM6045816 mrna_ORC4_14to17h_3
GSM6045817 mrna_ORC4_14to17h_4
GSM6045818 mrna_ORE50_ORC4_14to17h_1
GSM6045819 mrna_ORE50_ORC4_14to17h_2
GSM6045820 mrna_ORE50_ORC4_14to17h_3
GSM6045821 mrna_ORE50_ORC4_14to17h_4
GSM6045825 single cell RNAseq in 14 to 14.5h wildtype OregonR embryos using 10x Rep1
GSM6045826 single cell RNAseq in 14 to 14.5h wildtype OregonR embryos using 10x Rep2
GSM6045827 single cell RNAseq in 14 to 14.5h Lola-I mutant ORC4 embryos using 10x Rep1
GSM6045828 single cell RNAseq in 14 to 14.5h Lola-I mutant ORC4 embryos using 10x Rep2

Genome browser session
(e.g. UCSC)

All the tracks can be viewed by going to:

https://epigenomegateway.wustl.edu/browser/?genome=dm6&&position=chrX:10328379-10330370&hub=https://mitra.stanford.edu/kundaje/vir/lola_paper/WashU_all_load.json

Alternatively, specific files can be viewed by going to:

https://epigenomegateway.wustl.edu/browser/?genome=dm6&&position=chrX:10328379-10330370&hub=https://mitra.stanford.edu/kundaje/vir/lola_paper/WashU_all.json

and selecting individual files from Tracks>Custom track facet table

Methodology

Replicates

All experiments are performed in biological replicates, expect the following. MNAse 2-4 hrs dataset, which is part of a previously published dataset like many of the wild-type experiments, is not available as replicate. However, the results are consistent with other comparable datasets from other sources from the 2-4 hrs wild-type OregonR embryos.

Sequencing depth

GEO sample name total_reads experiment_type
Dme_emb_14-17h_Lola_I_1 16,230,383 ChIP-seq
Dme_emb_14-17h_Lola_I_2 14,400,374 ChIP-seq
Dme_emb_14-17h_Rpb3_1 26,938,616 ChIP-seq
Dme_emb_14-17h_Rpb3_2 23,949,792 ChIP-seq
Dme_emb_14-17h_Rpb3_3 44,306,640 ChIP-seq
Dme_emb_14-17h_WCE_1 36,951,883 ChIP-seq
Dme_emb_14-17h_WCE_2 17,235,131 ChIP-seq
Dme_emb_14-17h_WCE_3 6,372,194 ChIP-seq
Dme_emb_2-4h_Rpb3_1 24,040,990 ChIP-seq
Dme_emb_2-4h_Rpb3_2 58,069,075 ChIP-seq
Dme_emb_2-4h_Rpb3_3 57,043,990 ChIP-seq
Dme_emb_2-4h_WCE_1 26,357,554 ChIP-seq
Dme_emb_2-4h_WCE_2 3,875,394 ChIP-seq
Dme_emb_2-4h_WCE_3 3,445,079 ChIP-seq
ATAC-seq in Lola-I mutant ORC4 14-17hrs embryo Rep1 7,957,875 ATAC-seq
ATAC-seq in Lola-I mutant ORC4 14-17hrs embryo Rep2 40,426,508 ATAC-seq
ATAC-seq in Lola-I mutant ORC4 17-20hrs embryo Rep1 5,127,639 ATAC-seq
ATAC-seq in Lola-I mutant ORC4 17-20hrs embryo Rep2 5,048,219 ATAC-seq
dme_emb_act_Lola_I_orc4_14-17h_Rpb3_1 60,945,329 ChIP-seq
dme_emb_act_Lola_I_orc4_14-17h_Rpb3_2 52,549,530 ChIP-seq
dme_emb_act_Lola_I_orc4_14-17h_WCE_1 29,074,152 ChIP-seq
dme_emb_act_Lola_I_orc4_14-17h_WCE_2 29,990,394 ChIP-seq
ATAC-seq in Lola-I rescue 14-17hrs embryo Rep1 108,695,173 ATAC-seq
ATAC-seq in Lola-I rescue 14-17hrs embryo Rep2 59,654,676 ATAC-seq
Dme_emb_orc4_14-17h_Rpb3_1 23,326,995 ChIP-seq
Dme_emb_orc4_14-17h_Rpb3_2 48,802,173 ChIP-seq
Dme_emb_orc4_14-17h_Rpb3_3 40,449,532 ChIP-seq
Dme_emb_orc4_14-17h_WCE_1 17,001,884 ChIP-seq
Dme_emb_orc4_14-17h_WCE_2 9,050,039 ChIP-seq
Dme_emb_orc4_14-17h_WCE_3 7,779,274 ChIP-seq
ATAC-seq in Lola-I mutant transheterozygous ORE50/ORC4 14-17hrs embryo Rep1 22,274,807 ATAC-seq
ATAC-seq in Lola-I mutant transheterozygous ORE50/ORC4 14-17hrs embryo Rep2 16,518,498 ATAC-seq
ATAC-seq in wildtype OregonR 14-17hrs embryo Rep1 13,092,315 ATAC-seq
ATAC-seq in wildtype OregonR 14-17hrs embryo Rep2 31,276,353 ATAC-seq
ATAC-seq in wildtype INTACT Epidermis 14-17hrs embryo Rep1 21,264,570 ATAC-seq

ATAC-seq in wildtype INTACT Epidermis 14-17hrs embryo Rep2 36,336,644 ATAC-seq
ATAC-seq in wildtype INTACT Gut 14-17hrs embryo Rep1 6,623,886 ATAC-seq
ATAC-seq in wildtype INTACT Gut 14-17hrs embryo Rep2 55,259,604 ATAC-seq
ATAC-seq in wildtype INTACT Gut 14-17hrs embryo Rep3 50,996,861 ATAC-seq
ATAC-seq in Lola-I mutant ORC4 Gut 14-17hrs embryo Rep1 19,441,627 ATAC-seq
ATAC-seq in Lola-I mutant ORC4 Gut 14-17hrs embryo Rep2 37,665,649 ATAC-seq
Dme_MNase_14-17h_1 86,019,591 MNase-seq
Dme_MNase_14-17h_2 21,027,556 MNase-seq
Dme_MNase_2-4h_1 21,267,031 MNase-seq
Dme_MNase_orc4_14-17h_1 37,392,405 MNase-seq
Dme_MNase_orc4_14-17h_2 40,811,588 MNase-seq
ATAC-seq in wildtype INTACT Muscle 14-17hrs embryo Rep1 32,770,960 ATAC-seq
ATAC-seq in wildtype INTACT Muscle 14-17hrs embryo Rep2 24,987,222 ATAC-seq
ATAC-seq in wildtype INTACT Neuron 14-17hrs embryo Rep1 32,767,567 ATAC-seq
ATAC-seq in wildtype INTACT Neuron 14-17hrs embryo Rep2 27,033,154 ATAC-seq
ATAC-seq in wildtype INTACT Trachea 14-17hrs embryo Rep1 7,535,437 ATAC-seq
ATAC-seq in wildtype INTACT Trachea 14-17hrs embryo Rep2 43,641,789 ATAC-seq
ATAC-seq in wildtype INTACT Trachea 14-17hrs embryo Rep3 68,103,551 ATAC-seq
ATAC-seq in Lola-I mutant ORC4 Trachea 14-17hrs embryo Rep1 15,969,180 ATAC-seq
ATAC-seq in Lola-I mutant ORC4 Trachea 14-17hrs embryo Rep2 77,132,440 ATAC-seq
Epidermis_7021_INTACT_Lola_I_14-17h_1 14,235,851 ChIP-seq
Epidermis_7021_INTACT_Lola_I_14-17h_2 20,716,290 ChIP-seq
Epidermis_7021_INTACT_Lola_I_14-17h_3 17,266,609 ChIP-seq
Epidermis_7021_INTACT_Rpb3_14-17h_1 44,240,601 ChIP-seq
Epidermis_7021_INTACT_Rpb3_14-17h_2 36,891,685 ChIP-seq
Epidermis_7021_INTACT_WCE_14-17h_1 28,537,717 ChIP-seq
Epidermis_7021_INTACT_WCE_14-17h_2 60,230,563 ChIP-seq
Glia_repo_INTACT_Rpb3_14-17h_1 44,591,846 ChIP-seq
Glia_repo_INTACT_Rpb3_14-17h_2 24,841,699 ChIP-seq
Glia_repo_INTACT_WCE_14-17h_1 30,167,676 ChIP-seq
Glia_repo_INTACT_WCE_14-17h_2 35,369,724 ChIP-seq
Gut_110394_INTACT_Lola_I_14-17h_1 30,106,608 ChIP-seq
Gut_110394_INTACT_Lola_I_14-17h_2 25,748,692 ChIP-seq
Gut_110394_INTACT_Rpb3_14-17h_1 10,383,497 ChIP-seq
Gut_110394_INTACT_Rpb3_14-17h_2 29,045,287 ChIP-seq
Gut_110394_INTACT_WCE_14-17h_1 5,866,739 ChIP-seq
Gut_110394_INTACT_WCE_14-17h_2 35,463,852 ChIP-seq
Muscle_mef2_INTACT_Lola_I_14-17h_1 15,239,293 ChIP-seq
Muscle_mef2_INTACT_Lola_I_14-17h_2 24,745,248 ChIP-seq
Muscle_mef2_INTACT_Lola_I_14-17h_3 13,606,985 ChIP-seq
Muscle_mef2_INTACT_Rpb3_14-17h_1 22,371,281 ChIP-seq
Muscle_mef2_INTACT_Rpb3_14-17h_2 23,488,900 ChIP-seq
Muscle_mef2_INTACT_WCE_14-17h_1 14,927,266 ChIP-seq
Muscle_mef2_INTACT_WCE_14-17h_2 13,149,824 ChIP-seq
single cell RNAseq in 14 to 14.5h Lola-I mutant ORC4 embryos using 10x Rep1 367,563,025 scRNA-seq
single cell RNAseq in 14 to 14.5h Lola-I mutant ORC4 embryos using 10x Rep2 408,011,279 scRNA-seq
Neuron_elav_INTACT_Lola_I_14-17h_1 15,343,259 ChIP-seq
Neuron_elav_INTACT_Lola_I_14-17h_2 26,682,836 ChIP-seq
Neuron_elav_INTACT_Lola_I_14-17h_3 19,561,149 ChIP-seq
Neuron_elav_INTACT_Rpb3_14-17h_1 24,428,355 ChIP-seq
Neuron_elav_INTACT_Rpb3_14-17h_2 45,849,454 ChIP-seq
Neuron_elav_INTACT_WCE_14-17h_1 18,470,763 ChIP-seq
Neuron_elav_INTACT_WCE_14-17h_2 9,704,165 ChIP-seq
mrna_ORC4_14to17h_1 31,178,929 mrna-seq
mrna_ORC4_14to17h_2 29,479,138 mrna-seq
mrna_ORC4_14to17h_3 30,173,289 mrna-seq
mrna_ORC4_14to17h_4 29,867,637 mrna-seq
mrna_ORE50_ORC4_14to17h_1 12,596,043 mrna-seq
mrna_ORE50_ORC4_14to17h_2 13,746,929 mrna-seq
mrna_ORE50_ORC4_14to17h_3 9,420,118 mrna-seq
mrna_ORE50_ORC4_14to17h_4 11,266,165 mrna-seq
Trachea_8807_INTACT_Lola_I_14-17h_1 43,764,289 ChIP-seq
Trachea_8807_INTACT_Lola_I_14-17h_2 17,026,413 ChIP-seq
Trachea_8807_INTACT_Rpb3_14-17h_1 33,027,501 ChIP-seq
Trachea_8807_INTACT_Rpb3_14-17h_2 30,404,869 ChIP-seq
Trachea_8807_INTACT_WCE_14-17h_1 20,379,348 ChIP-seq
Trachea_8807_INTACT_WCE_14-17h_2 22,664,022 ChIP-seq
Trachea_8807_orc4_INTACT_Rpb3_14-17h_1 77,874,850 ChIP-seq
single cell RNAseq in 14 to 14.5h wildtype OregonR embryos using 10x Rep1 359,279,381 scRNA-seq
single cell RNAseq in 14 to 14.5h wildtype OregonR embryos using 10x Rep2 394,597,861 scRNA-seq
mrna_wt_14to17h_1 31,250,307 mrna-seq
mrna_wt_14to17h_2 31,430,735 mrna-seq
mrna_wt_14to17h_3 35,407,625 mrna-seq
mrna_wt_14to17h_5 10,844,925 mrna-seq
mrna_wt_14to17h_6 10,649,320 mrna-seq
mrna_wt_2to4h_1 34,071,228 mrna-seq

mrna_wt_2to4h_2 16,125,416 mrna-seq
mrna_wt_2to4h_3 34,985,169 mrna-seq

Antibodies

Lola-I (custom-made by Genescript), Pol II (Rpb3, custom made from GeneScript, Zeitlinger lab 163185-50)

Peak calling parameters

Peaks were called using MACS version 2.1.2 using the command line function "callpeak -g dm --keep-dup all -n lola_i_peaks_1 -t Dme_emb_14-17h_Lola_I_1.bam -c Dme_emb_14-17h_WCE_1.bam -p 1e-5"

Data quality

Data quality is confirmed by replicate correlations as shown in Supplementary Data 5. Peak calling is only applicable to the Lola-I dataset. To be conservative, only the top 1000 peaks present in both Lola-I ChIP-seq replicates were used as Lola-I peaks.

Software

The analysis code required to process the raw files and to perform the additional biological analysis are available on GitHub at https://github.com/zeitlingerlab/Ramalingam_Lola_2022.git In addition the Amazon Machine Image (ami-id: ami-013fa11a4a9b52628)available through the amazon cloud provides analysis code, the raw data, the processed data, and all the required software in a pre-installed way.

Briefly the following software was used:
ChIP-seq data and ATAC-seq experiments were aligned using Bowtie (1.1.2)
Bulk mRNAseq samples, pseudo-alignment was performed using the Kallisto package (0.46.0)

scRNA-seq samples, alignment and separations of reads from different cells and quantification of gene expression were done using the Cell Ranger pipeline (2.1.1)

The secondary biological analysis is done in R (3.6.1) using publicly available libraries. For more details on individual analysis, https://github.com/zeitlingerlab/Ramalingam_Lola_2022.git
